# Endoscope-Assisted Combined Supracerebellar Infratentorial and Endoscopic Transventricular Approach to the Pineal Region: A Technical Note

**DOI:** 10.7759/cureus.520

**Published:** 2016-03-06

**Authors:** Daniel Felbaum, Hasan R Syed, Joshua E Ryan, Walter C Jean, Amjad Anaizi

**Affiliations:** 1 Neurosurgery, Medstar Georgetown University Hospital

**Keywords:** skull base, endoscope skull base, pineal tumor, glioma surgery

## Abstract

Neoplasms of the pineal region comprise less than 2% of all intracranial lesions. A variety of techniques have been adapted to gain access to the pineal region. Classic approaches employ the use of the microscope. More recently, the endoscope has been utilized to improve access to such deep-seated lesions.

A 62-year-old female presented with a heterogeneously enhancing lesion in the pineal region with associated hydrocephalus. On exam, the patient exhibited Parinaud’s syndrome. The patient initially underwent a single burr hole endoscopic third ventriculostomy and biopsy of the lesion. Initial pathology was consistent with a grade III astrocytoma. Following a period of recuperation, she returned for definitive surgical resection.

A suboccipital craniectomy was performed in the sitting position. Prior to dural opening, an endoscope was inserted into the right lateral ventricle through the prior burr hole.The endoscope was passed through the foramen of Monro and the tumor could be visualized along the posterior third ventricle. The patient underwent a standard supracerebellar infratentorial approach aided by the microscope. After initial debulking of the pineal lesion, an endoscope was utilized to guide the depth of resection and assist in dissection with transventricular manipulation of the tumor. During the final stages of resection from the craniotomy, the endoscope was used to help visualize the posterior supracerebellar corridor. This assisted in the assessment of the extent of resection. The endoscope was also utilized for the removal of intraventricular blood products following tumor resection.

The patient was extubated and transferred to the intensive care unit. A postoperative contrast-enhanced magnetic resonance imaging (MRI) revealed greater than 95% resection, with expected residual within the midbrain.

The combined supracerebellar infratentorial and transventricular endoscope-assisted approach provided maximum visualization and aided in optimal resection of a traditionally difficult pineal region tumor. Further experience with this combined technique may allow for improved surgical outcomes for these complex lesions.

## Introduction

A variety of pathological entities may be encountered within the pineal region [1]. Traditionally, approaches to the pineal region employ corridors that allow access to a difficult area utilizing microsurgical technique. Each microsurgical technique has unique considerations to achieve maximal exposure with minimal morbidity. These open or endoscope-assisted, microsurgical techniques include: occipital transtentorial, midline or paramedian supracerebellar infratentorial (SCIT), combined supra- and infratentorial approach, or posterior transventricular approach [2-5]. 

More recent improvements in endoscopic technology have allowed neurosurgeons to expand the armamentarium in tackling difficult to access pineal lesions [5]. In the available literature, endoscopic techniques to the pineal region remain limited to smaller case series. We attempt to add to the body of literature by reporting a combined supra- and infratentorial approach to the pineal region with the utilization of both the endoscope and microscope to improve surgical morbidity.

## Technical report

### Case report

**Figure 1:**
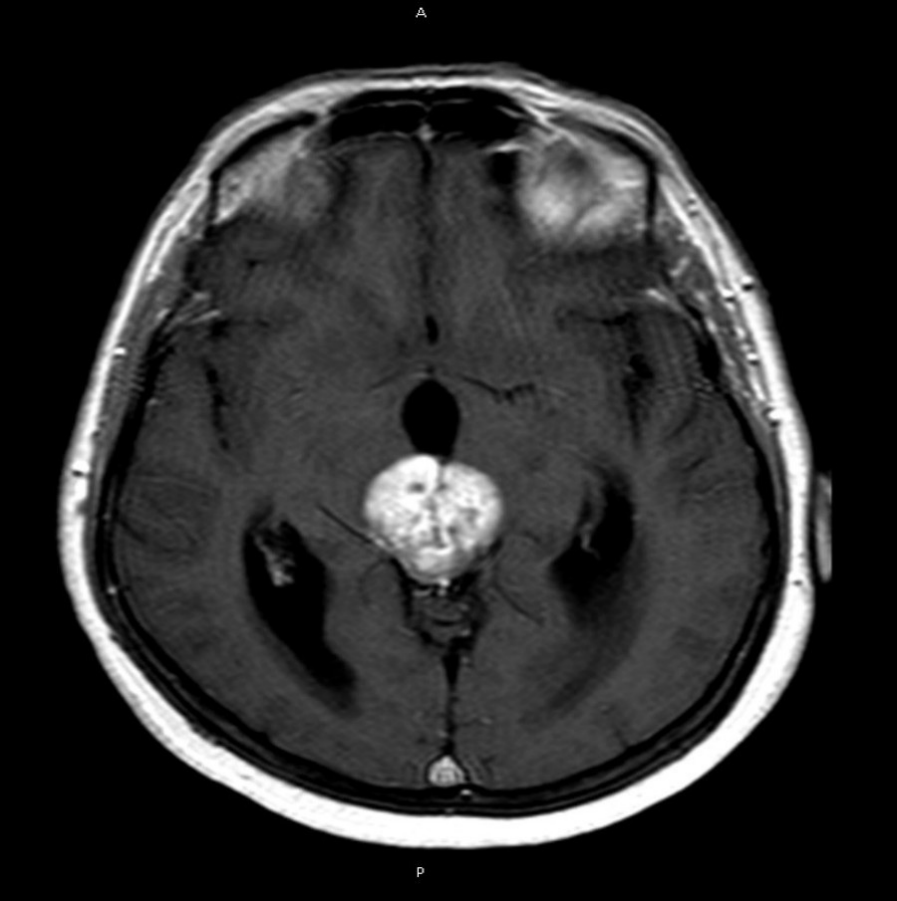
Preoperative MRI Preoperative axial MRI with contrast showing an enhancing pineal region lesion in the center of the picture.

**Figure 2:**
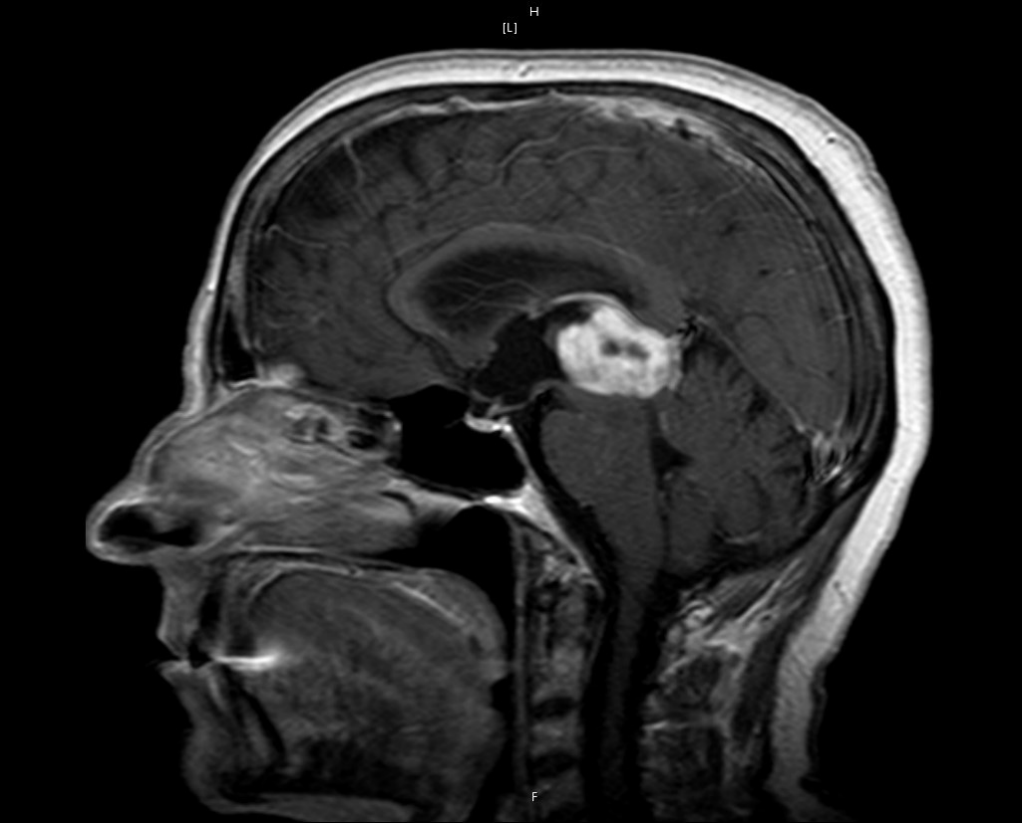
Preoperative MRI Sagittal MRI with contrast revealing an enhancing pineal region lesion suspicious for a glial-type lesion.

A 62-year-old female presented to the neurosurgical service with a newly diagnosed pineal region lesion. The post-contrast MRI revealed a heterogeneously enhancing lesion that appeared intrinsic to the pineal gland (Figures 1, 2).

Using previously reported techniques, the patient underwent an endoscopic third ventriculostomy (ETV) with concomitant biopsy to attain pathologic diagnosis and to treat her triventricular hydrocephalus. The hydrocephalus did resolve and her headaches and Parinaud's improved, but did not resolve despite high dose steroids. Following a confirmed diagnosis of a high grade glioma (minimum grade III), and extensive discussion with the patient and her family, the patient was returned to the operating theater for more complete resection.

### Operative technique

**Figure 3:**
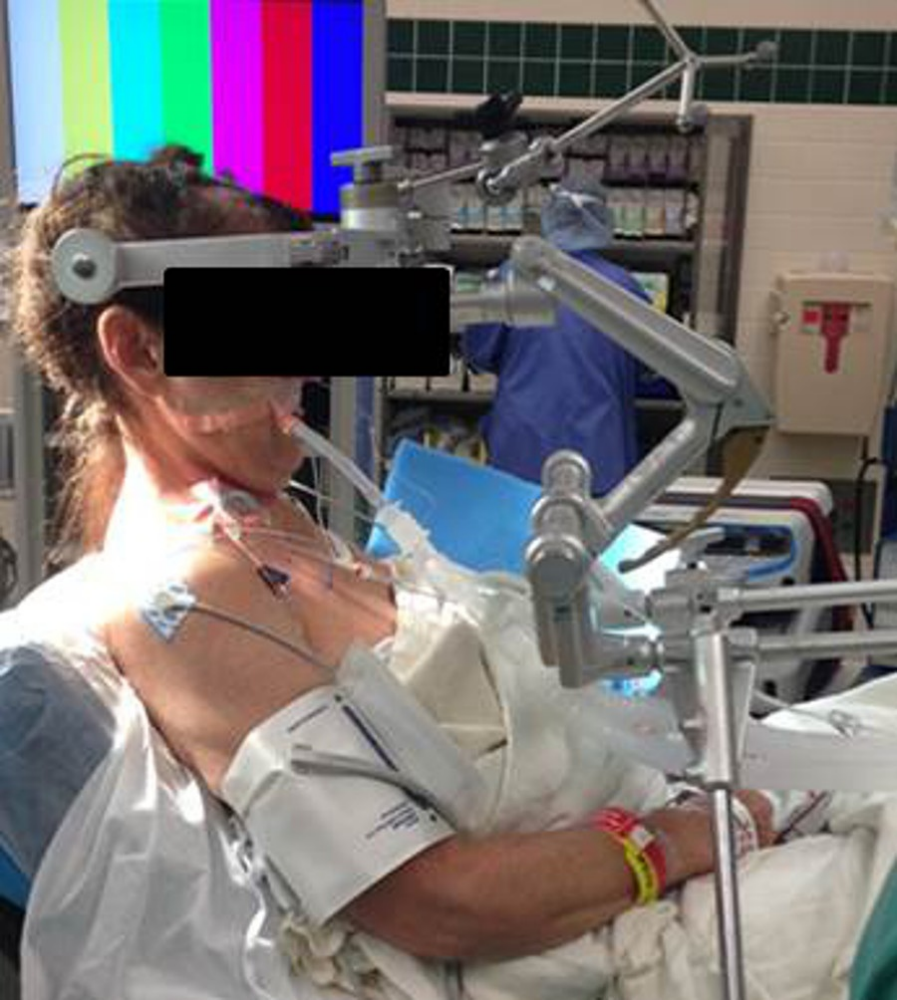
Intraoperative set-up Patient in the sitting craniotomy position to allow access for both approaches via the infratentorial corridor and the transventricular corridor.

**Figure 4:**
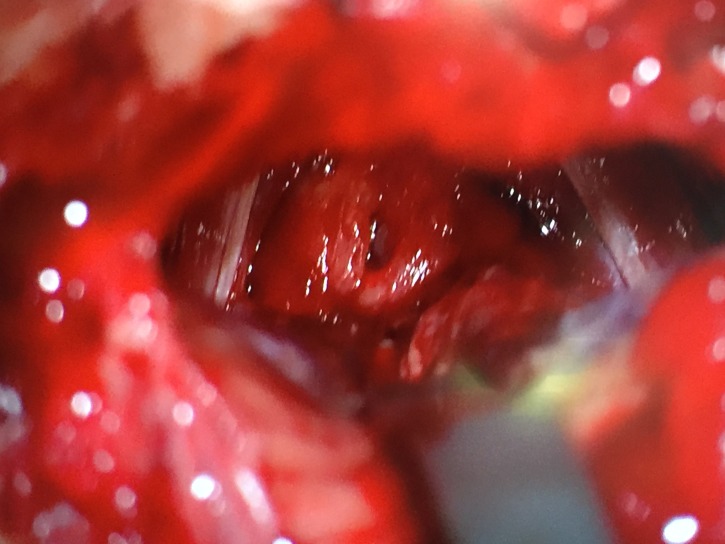
Operative view through microscope Intraoperative microscope-assisted photograph detailing the pineal tumor in the center and the deep venous structures surrounding the tumor.

Written informed consent was obtained prior to surgery. After induction of adequate anesthesia, a central venous catheter and precordial Dopplers were placed. The patient’s central venous pressure was insured to be normal. The patient was placed in the sitting position in preparation for a supracerebellar infratentorial (SCIT) open microsurgical craniotomy (Figures 3, 4).

**Figure 5:**
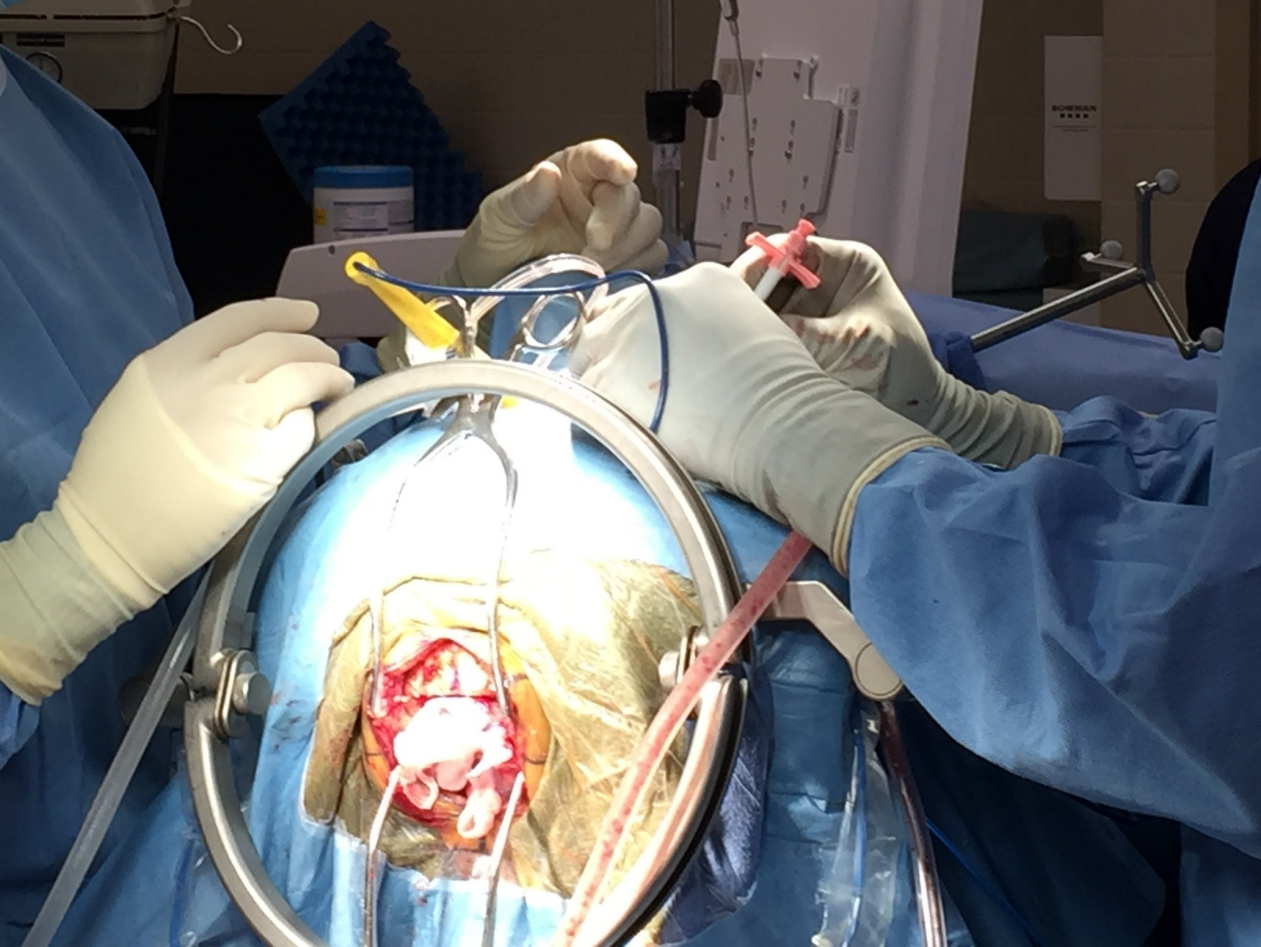
Operative corridor through transventricular endoscope-assisted approach Intraoperative photograph showing endoscope sheath placement to gain access to the right frontal ventricle. The craniotomy performed for the supracerebellar infratentorial approach is seen in the inferior aspect of the picture.

After completing the craniotomy the patient’s previous burr hole for ETV was accessed for insertion of the endoscopes (Figure 5).

The lesion invading the posterior wall of the third ventricle was visualized and the rigid endoscope ( LOTTA® Endoscope, Storz, Germany) securely docked. 

**Figure 6:**
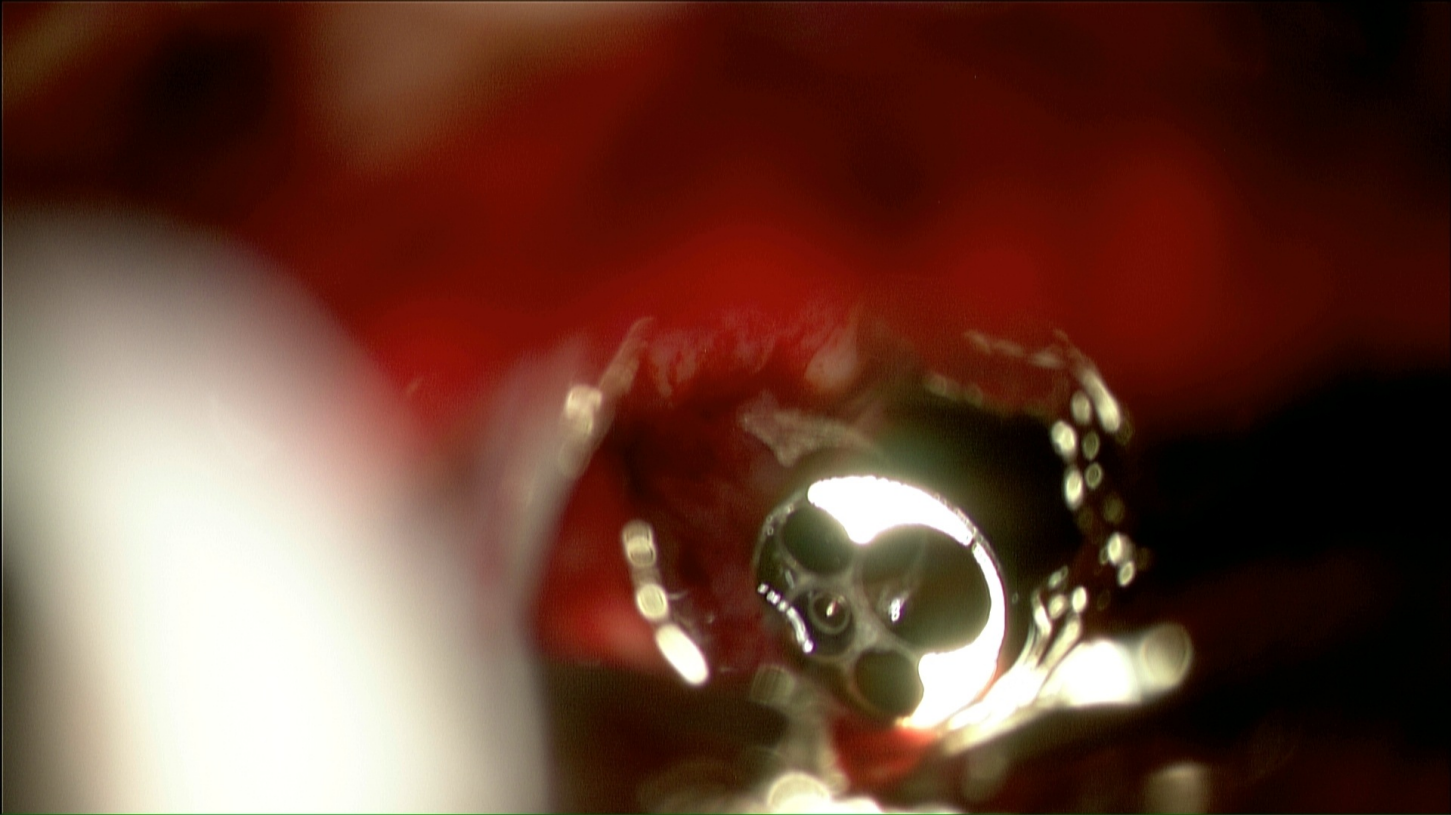
Intraoperative photo using the microscope in the posterior fossa Microscope-assisted image of the endoscope, sitting in the third ventricle, aiding extent of resection.

Using standard microsurgical technique a supracerebellar infratentorial corridor was created. The pineal tumor was debulked in the standard fashion. The basal vein of Rosenthal and internal cerebral veins were identified and fastidiously preserved through the resection. As the resection proceeded anteriorly, the light of the endoscope was used to guide the direction and limits of the microsurgical resection. The endoscopic transventricular approach was utilized to push the tumor posteriorly towards the infratentorial exposure. Upon reaching the distal portion of the infratentorial approach and accessing the posterior wall of the third ventricle, the endoscope was encountered. This provided further assurance marking the boundaries for total resection (Figure 6).

The rigid endoscope was then removed from the ventricle and utilizing a two-surgeon technique, used to inspect the resection cavity through the infratentorial corridor. The craniotomy was then closed in the standard manner, and an external ventricular drain (EVD) was placed in the right lateral ventricle.

### Postoperative course

**Figure 7:**
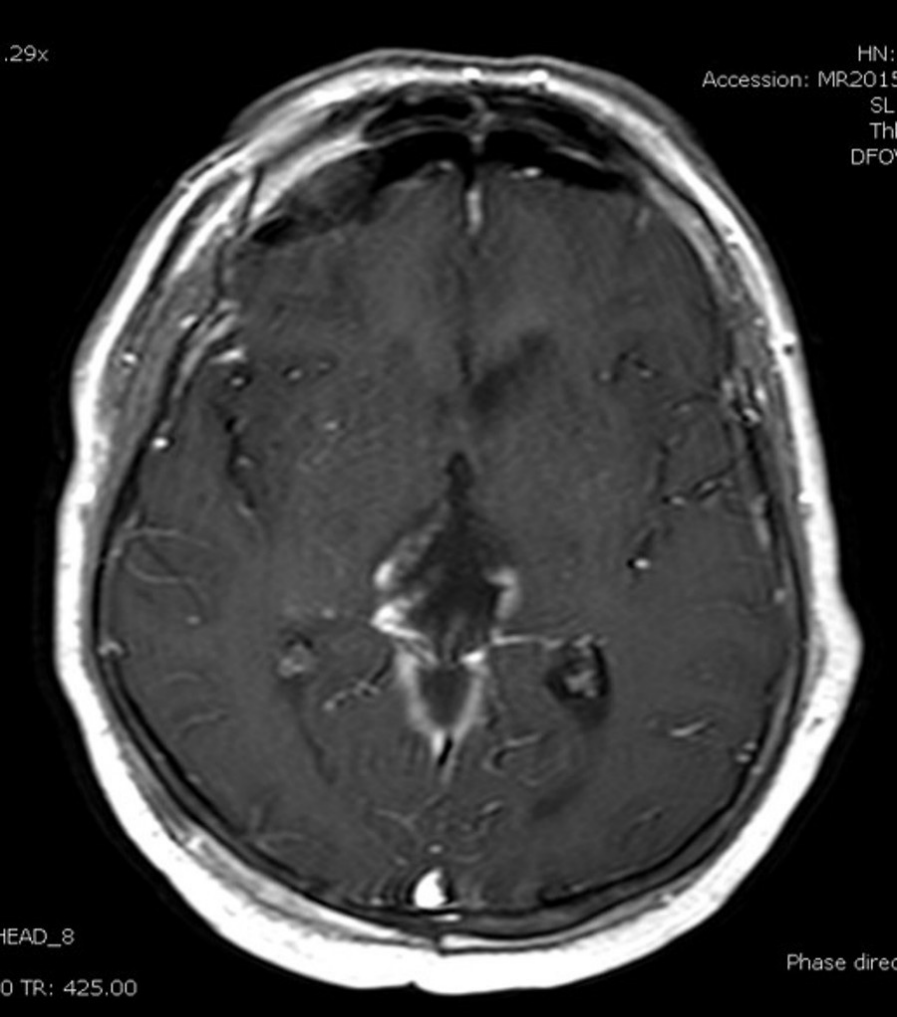
Postoperative MRI with contrast Postoperative T1 MRI with contrast showing close to gross total resection of the pineal glioma as compared to Figure 1.

The patient’s MRI confirmed a greater than 95% resection, with preservation of the deep venous system (Figure 7).

The EVD was kept clamped and removed as the patient’s neurological exam improved. The hospital course was complicated by a postoperative ventriculitis that was treated with long-term antibiotics. At discharge, the patient’s exam was a Glasgow Coma Score (GCS) 15 with a modified Rankin Score (mRS) of 2.

## Discussion

A plethora of pathology can afflict the pineal region [1]. More commonly, these lesions can be classified as: germ cell tumors, non-germ cell tumors, pineal parenchymal lesions, and pineal region gliomas. In most instances, an accepted treatment paradigm for these lesions involve stereotactic or open biopsy and cerebrospinal fluid version via endoscopic third ventriculostomy. More recently, pineal region lesions are being evaluated in a single operative session via endoscopic third ventriculostomy with stereotactic biopsy. This provides pathologic tissue diagnosis and obviates the need of a ventriculoperitoneal shunt. As a result, this allows for identification of lesions that may be sensitive to chemotherapy or radiation therapy and negate further surgical involvement. In addition, cerebrospinal fluid (CSF) markers such as b-HCG, AFP, and cytology can be analyzed to further aid in diagnosis [2-3, 6-7].

Gliomas of the pineal region constitute approximately 15% of histopathology. More specifically, high grade gliomas (WHO grade III or IV) of the pineal region are even more rare. In a comprehensive meta-analysis of 683 patients with pineal gliomas, 349 were with a defined histotype, anaplastic astrocytoma was present in 32 patients and glioblastoma multiforme was present in 65 patients [8]. As expected for rare lesions, the treatment modalities presented are varied. Ultimately, higher grade pineal gliomas are treated in a similar fashion to more commonly found supratentorial high grade lesions. In these cases, the patients undergo maximal safe resection, followed by chemotherapy and radiation [8]. The prognoses for these lesions are similarly poor, with the available information on grade III astrocytomas having an average follow-up of 14 months. In addition, it is important to attempt to separate pineal gliomas from tectal gliomas, which are geographically close, but have a more benign clinical course [6]. In our patient with a good functional status and a high grade lesion, the decision to attain maximum safe resection was performed to provide a longer lasting survival benefit, and provide relief from brainstem compression.

Surgery of pineal region pathology is difficult due to the limited access in a small and deep corridor. This is highlighted by the numerous approaches that can be utilized to performed microsurgery in this region [2-3, 9-11]. The literature has established benefits and limitations for each approach. In addition, the rarity of these lesions can make operations in this region daunting. The advent of microsurgical technique has allowed for reduced morbidity in the surgical treatment of these lesions. More recently, there is interest in applying endoscopic techniques to improve surgeon comfort and technique to this region [12-14]. In particular, Uschold et al. report on nine consecutive patients who underwent endoscopic assisted SCIT for resection of pineal region pathology. Using a two-surgeon, three-hand technique, avoiding a bimanual technique, they achieved maximal safe resection without surgical complications. Furthermore, they conclude that the endoscope provided improved visualization and surgical maneuverability in comparison to their experience using the microscope. More importantly, patients remained or had improved functional outcome at latest follow-up. In the same article, the authors advocate for a “posterior ETV,” as well as an endoscope-assisted biopsy from a posterior approach. In the article, they argue given the possibility of sampling error as high as 70%, a posterior paramedian endoscopic approach using microsurgical technique may allow for improved visualization, accessing the posterior third ventricle, and attaining a better tissue diagnosis. Our technique is different because of the marriage of both approaches, endoscopic transventricula and open craniotomy via supracerebellar infratentorial approach, rather than a single separate corridor or modality approach.

We believe our technical report adds to the increasing body of literature regarding endoscopic uses for pineal region tumors. This technique is unique and advantageous in that it allows for tumor resection and manipulation while visualizing both the posterior and anterior borders of the tumor throughout the operation. The combined microsurgical and endoscopic technique allowed us to safely determine the extent of our resection, transventricular tumor manipulation, and guided the direction of the microsurgical portion of the procedure. At the conclusion of the resection, the endoscope use through both the supratentorial and infratentorial approaches, further aided in confirming the completeness of resection in a less surgeon obtrusive manner. An argument against this technical report is the need for a separate surgical site, which may increase the morbidity of the procedure. A weakness of this approach is the need for a two-surgeon technique. The risk for postoperative ventriculitis may also be increased secondary to the re-accessing of the ventricle and prolonged exposure to the endoscope. In addition, prolonged traction on the fornix may increase memory dysfunction. We believe this technique has the potential to improve outcomes in the surgical treatment of these difficult pineal region lesions. Further experience with this unique surgical technique is necessary.

## Conclusions

We describe the first account of a combined supracerebellar infratentorial and transventricular endoscope-assisted approach to the pineal region. This technique provided maximum safe resection and confirmed the feasibility of a multiple corridor approach to the skull base. Further experience with this combined technique may allow for improved surgical outcomes for these complex regional tumors.
